# Intraoperative Ultrasound-Guided Transversus Abdominis Plane Catheters Placed for Post-operative Analgesia Following Pedicled Transverse Rectus Abdominis Myocutaneous Flap Breast Reconstruction: A Case Report

**DOI:** 10.7759/cureus.39045

**Published:** 2023-05-15

**Authors:** Richard M Missett, Sanaz Beig Zali, Jonathan Winograd, Pascal Scemama de Gialluly, A. Sassan Sabouri

**Affiliations:** 1 Anesthesiology, Children's Hospital of Philadelphia, Philadelphia, USA; 2 Anesthesiology, Massachusetts General Hospital, Boston, USA; 3 Plastic and Reconstructive Surgery, Massachusetts General Hospital, Boston, USA; 4 Anesthesiology, University of Massachusetts, Worcester, USA

**Keywords:** transverse rectus abdominis myocutaneous flap, breast reconstruction, transversus abdominis plane catheter, opioid-sparing techniques, regional anesthesia

## Abstract

Transverse rectus abdominis (TRAM) flap reconstruction of the breast is a procedure in which a flap of skin, fat, and underlying rectus abdominis muscle is used to reconstruct the breast. This procedure is commonly performed after mastectomy and results in significant pain at the donor abdominal site. We present this case of a 50-year-old female undergoing pedicled TRAM flap surgery in which ultrasound-guided transversus abdominis plane (TAP) catheters were placed intraoperatively, in a novel fashion: under ultrasound guidance, directly on the abdominal musculature, without overlying fat, subcutaneous tissue, or dressing. Our case-reported numeric pain scores ranged from 0-5/10 during postoperative days one to two. The patient's IV morphine requirement on postoperative days zero to two ranged between 1.34 mg to 2.6 mg per day, representing a significant decrease compared to literature-reported opioid consumption after such surgery. Her pain and opioid consumption increased significantly after catheter removal, suggesting the efficacy of our intraoperative TAP catheters.

## Introduction

Pedicled transverse rectus abdominis muscle (TRAM) flap reconstruction of the breast is a procedure in which a flap of skin, fat, and underlying rectus abdominis muscle is used to reconstruct the breast [1]. This reconstructive approach is commonly used after mastectomy. The procedure involves a large lower abdominal incision extending from both flanks between the umbilicus and suprapubic area. After releasing the rectus abdominis muscle and preserving its superior vascular bundle, the flap is typically tunneled to the mastectomy pocket. The blood supply to the flap is provided from the superior epigastric artery and vein.

TRAM flaps are associated with significant abdominal pain due to extensive skin and superficial muscle incisions and tension due to flap tunneling and transposition [2-4]. As a result, and despite their advantages, effective pain management remains a significant drawback for this method [4]. Varying methods have been used for postoperative pain control, such as patient-controlled analgesia, epidural catheters, continuous wound instillation with local anesthetics, quadratus lumborum blocks or catheters, single-shot transversus abdominis plane (TAP) blocks, and erector spinae plane block (ESPB). Each approach is associated with limitations [2-14].

Out of the above methods, TAP blocks have shown many advantages, including reductions in opioid utilization and hospital stay times in different case studies and meta-analyses [12-14]. Furthermore, catheter-based regional techniques provide continuous, extended analgesia for postoperative pain; however, postoperative catheter placement may be difficult due to lateral extension of the incision, which may be overlaid by surgical dressing. TAP blocks/catheters delivered through surgically exposed abdominal musculature are one solution to this barrier. This has been reported for other similar reconstructive procedures, such as deep inferior epigastric perforator (DIEP) flap surgery [8].

In this paper, we discuss a pedicled TRAM flap in which TAP catheters were placed intraoperatively under ultrasound guidance by the regional anesthesiologist in a patient who had a known sensitivity to narcotics. We hypothesize that with ultrasound guidance, TAP catheters could be placed precisely in the correct muscle plane, be used immediately, provide excellent postoperative pain control and reduce postoperative opioid consumption.

## Case presentation

A 50-year-old, 69-kilogram (BMI 27) female with a past medical history of left breast ductal carcinoma in situ who underwent mastectomy eight years prior presented for left-sided TRAM flap reconstruction. She had intolerances to fentanyl, which caused severe nausea, and oxycodone, which caused itching.

The pedicled TRAM flap was performed under general endotracheal anesthesia. In addition to the inhaled anesthetic, she received a 1 mg/kg intravenous bolus of ketamine followed by an infusion at 5 mcg/kg/minute. She also received a total of 1.3 mg of intravenous hydromorphone in divided doses, with the last 0.3 mg given one hour prior to emergence. Ketamine was stopped approximately one hour before extubation. Approximately two and a half hours after surgery started, while the TRAM flap was being sewn to the pectoral muscles by the plastic surgery team, the TAP catheters were inserted by the regional anesthesiologist.

**Figure 1:**
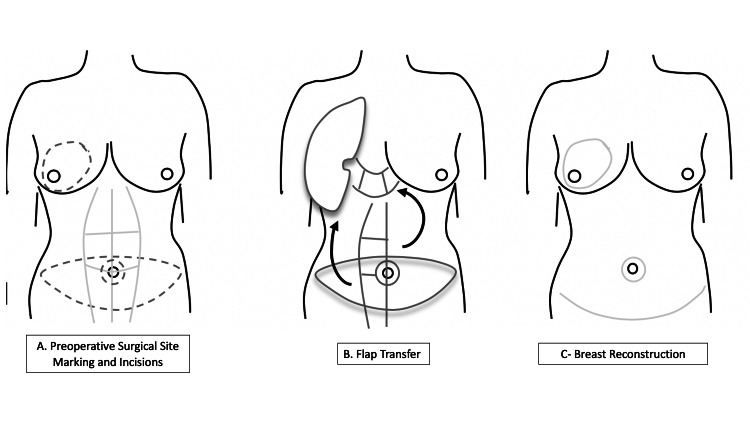
The pedicled TRAM flap process involving three different stages A) A large lower abdominal incision and release of rectus muscle with the division of the inferior and preservation of superior vascular supply. B) Transfer of flap to the mastectomy pocket. C) TRAM flap inset and closure TRAM - transverse rectus abdominis muscle

**Figure 2:**
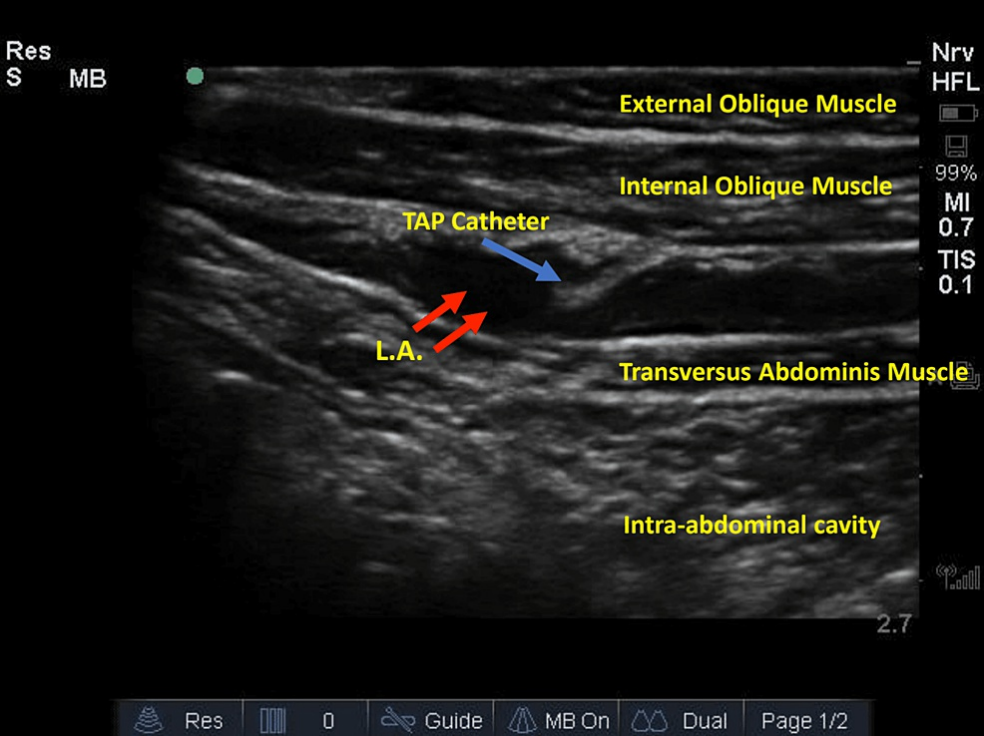
Ultrasonic anatomy and for the placement of TAP catheters done directly on abdominal musculature. No skin or subcutaneous tissue can be appreciated. The blue arrow shows the TAP catheter, and the red arrows show the spread of local anesthetic in the plane between transversus abdominis and internal oblique muscles. TAP -  transversus abdominis plane, LA - local anesthetic

The TAP catheters were inserted in a sterile fashion, directly over the external oblique muscle at the level of the umbilicus in the most lateral edge of the abdominal incision. Under ultrasound verification, three muscle layers: external oblique, internal oblique, and transversus abdominis were identified with no overlying skin and subcutaneous fat or tissue (Figure 1). An Arrow® StimuCath® 20 Ga. x 60 cm stimulating continuous peripheral nerve block catheter (Teleflex® North America, Morrisville, North Carolina) was advanced through a 19-gauge Tuohy needle under direct ultrasonic visualization using an in-plane approach (GE LOGIQTM Ultrasound Linear Probe 6-15 MHz, Milwaukee, Wisconsin). The needle was advanced into the plane between the internal oblique and transversus abdominis muscles, and normal saline was used to dissect the muscle planes and confirm the proper needle placement. A catheter was then advanced into the plane between the internal oblique and transversus abdominis plane and confirmed ultrasonically (Figure 2). The process was repeated on the contralateral side. Intraoperatively, each catheter was bolused with 20 mL of 0.25% bupivacaine.

**Figure 3:**
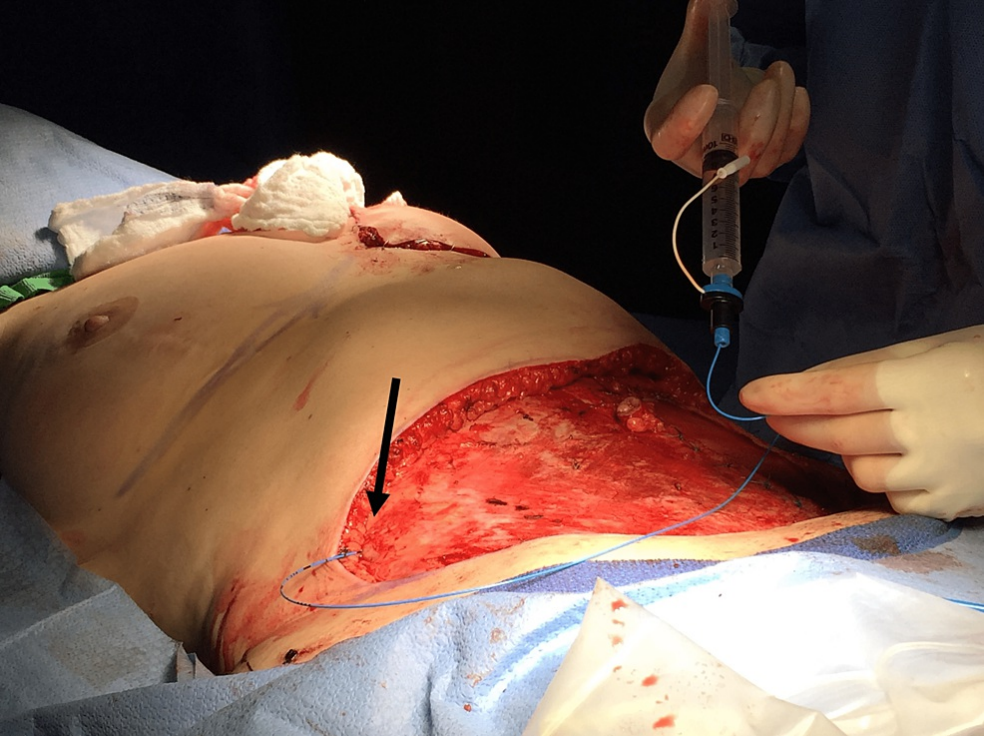
Placement of TAP catheter directly on the external oblique fascia (black arrow). Fixation of the catheter was performed by the surgical team at the end of the procedure. TAP -  transversus abdominis plane

The catheters exited laterally in the abdominal incision and were secured by the surgeon (Figure 3). The patient was extubated and taken to the post-anesthesia care unit (PACU). Both catheters were continuously infused with 0.1% bupivacaine at 8 mL/hr. The patient received one dose of 0.5 mg intravenous hydromorphone in the PACU. She did not require any additional intravenous narcotics throughout her admission. She began taking oral hydromorphone prior to her discharge from the PACU and received a total of 4 mg on postoperative day (POD) 0. Non-opioid adjuncts included acetaminophen, butalbital-acetaminophen-caffeine, gabapentin, and ibuprofen.

The TAP catheters were continued at 8 mL per hour per side. Her numeric pain scores ranged from 0-5/10 on POD 0. On POD one and two, the patient received 8 mg and 4 mg of oral hydromorphone, respectively. The patient's increased dose of oral hydromorphone on POD one was associated with pain during ambulation. Her pain was primarily located in the upper abdomen and lower chest. Numeric pain scores ranged from 2-4/10 on POD one and 2-3/10 on POD two.

On POD three, the TAP catheters were removed. Following the removal of the catheters, the patient's oral hydromorphone was switched to oral hydrocodone, of which she required 20 mg on POD three. She was discharged home on POD three with a prescription for hydrocodone/ acetaminophen 325/5 mg q6h as needed for pain. The patient reported good subjective pain control throughout her admission.

## Discussion

We described the successful placement of ultrasound-guided TAP catheters for postoperative pain control, performed directly on the abdominal wall musculature during pedicled TRAM flap reconstruction of the breast, contributing to a reduction in postoperative narcotic consumption. Mobilization of the TRAM flap results in the dissection and division of the intercostal nerves at multiple levels [3]. Therefore, the abdominal donor site is a major source of postoperative pain. Conventional postoperative pain relief protocols are primarily opioid-based, with associated side effects, such as constipation, sedation, nausea, respiratory depression, and the risk of dependency. Epidural catheters have been used to provide postoperative analgesia in patients undergoing TRAM flap reconstruction; however, urinary retention and interference with patients' mobilization are disadvantages [8,11,15,16]. 

Inter-fascial blocks such as quadratus lumborum (QL) blocks or catheters have been used for pain control for TRAM flap procedures. Nevertheless, they do not eliminate patients' need for rescue intravenous opioids [7]. QL or TAP blocks do not cover the muscular flap insertion site or the thoracic incisions, both of which may be a source of pain or discomfort [6,7].

The reported total morphine equivalent doses for pedicled TRAM flaps without TAP blocks in the literature range from approximately 70 to 80 mg on average [2,17,18]. On POD 0, our patient required a total of 4 mg oral hydromorphone, equivalent to 1.34 mg of intravenous morphine - 0.02 mg/kg/day for this 65 kg patient [19]. Her narcotic usage increased to 2.6 mg of intravenous morphine equivalents on POD one (0.04 mg/kg/day) and fell to 1.34 mg of intravenous morphine equivalents (0.02 mg/kg/day) on POD two. After the removal of the catheters on POD three, her narcotic requirement increased significantly to 6.7 mg of intravenous morphine equivalents (0.1 mg/kg/day) despite the passage of three days. The total intra- and postoperative opioid usage was approximately equivalent to 12.4 mg of morphine, suggesting the efficacy of the TAP catheters in reducing opioid consumption. The reported postoperative daily per kilogram morphine requirement for free TRAM flaps was 1.65 mg/kg/day. Although, due to the differences between the free and pedicled TRAM flaps, the comparability might be limited, we did see a substantial decrease in this regard as well [3].

Hivelin et al. found in a prospective study that bilateral ultrasound-guided TAP block after breast reconstruction by DIEP flap reduces but does not eliminate the interval and cumulative morphine requirements for the first 24 and 48 postoperative hours [8]. This study suggested that a single-shot TAP block with coverage limited to T10-L1 may not eliminate pain, especially since donor-recipient procedures require two operative areas - the abdominal donor and breast recipient sites [6]. Furthermore, the TAP blocks in this study were done by the surgical team, who may have limited experience performing such procedures. The study addressed the difficulty of doing TAP blocks on an open abdomen through an extended incision. It was the first example in the literature examining the performance of a single-shot TAP block directly on the abdominal wall musculature.

In our case, placing the TAP catheters after mobilization of the donor flap and before the closure of the abdominal site had the following benefits: 1) Improved visualization of the anatomy as the removal of the skin and subcutaneous tissue improved the visualization of abdominal planes under ultrasound (Figure 2). 2) Better exposure to the patient, unhindered by surgical dressings, due to performing the block intra-operatively before surgical closure. 3) Immediate postoperative pain control using the TAP catheters. 4) Earlier performance of the block by regional anesthesia-capable staff who may not be available later in the day, as these surgeries are often lengthy. 5) Decreased consumption of opioids for postoperative analgesia - the first dose of opioid pain medication was given four hours after completion of the TAP catheters, with the major source of the patient's pain at the flap insertion site. 6) Concurrent work with the surgical team, as the catheter placement on the abdominal site was performed while the surgical team was working on the thoracic component, reducing surgical and anesthesia time. Although we did not make such comparisons, future studies could investigate this technique's contribution to length of stay reduction, chronic opioid usage, and decreased cost of care.

As previously mentioned, continuous TAP blocks will not cover the muscular flap insertion site or the thoracic incisions following TRAM flap surgery. Therefore, combining this block with thoracic paravertebral blocks may improve overall analgesia; however, multifocal regional anesthesia raises concerns about the total dose of local anesthetic administered.

## Conclusions

In conclusion, this report described the placement of bilateral TAP catheters, performed intraoperatively, directly on surgically exposed abdominal wall musculature during pedicled TRAM flap reconstruction of the breast. This intervention contributed to a significant reduction in the patient's post-operative narcotic requirements. Although a randomized controlled trial is needed to further elucidate the effects of continuous TAP blockade done under direct visualization at the abdominal donor site for TRAM flap reconstruction of the breast, this case report clearly showed an effective intraoperative regional anesthetic technique to reduce narcotic usage.
